# Supplementary material for: MetaRibo-Seq measures translation in microbiomes
Source: Nat Commun. 2020 Jun 29;11:3268. doi: 10.1038/s41467-020-17081-z (PMC7324362; doi:10.1038/s41467-020-17081-z)
Supplement: Supplementary file 10 — Supplementary Data 7 [file 41467_2020_17081_MOESM10_ESM.zip › File2/Confidence_VeryHigh_Taxonomy/46073_out.krona.html]

Javascript must be enabled to view this page.

members
magnitude
magnitudeUnassigned
count
unassigned
taxon
rank

46073\_out

4

superkingdom
4
2

4
phylum
1239

4
class
186801

4
order
186802

family
2
186803

genus
1
841

species
1

SRS077641\_contig\_number\_23239
2292065

1407607
genus
1


SRS1041141\_contig\_number\_15157
1150298
species
1

1
family
31979

genus
1
1485

SRS050998\_contig\_number\_14080

1
species
1898207

SRS014459\_contig\_number\_610
